# Estetrol Modulates Endothelial Nitric Oxide Synthesis in Human Endothelial Cells

**DOI:** 10.3389/fendo.2015.00111

**Published:** 2015-07-22

**Authors:** Maria Magdalena Montt-Guevara, Maria Silvia Giretti, Eleonora Russo, Andrea Giannini, Paolo Mannella, Andrea Riccardo Genazzani, Alessandro David Genazzani, Tommaso Simoncini

**Affiliations:** ^1^Molecular and Cellular Gynecological Endocrinology Laboratory (MCGEL), Department of Clinical and Experimental Medicine, University of Pisa, Pisa, Italy; ^2^Department of Obstetrics and Gynecology, University of Modena and Reggio Emilia, Modena, Italy

**Keywords:** estetrol, estrogen, endothelial cells, nitric oxide, endothelial nitric oxide synthase

## Abstract

Estetrol (E4) is a natural human estrogen that is present at high concentrations during pregnancy. E4 has been reported to act as an endogenous estrogen receptor modulator, exerting estrogenic actions on the endometrium or the central nervous system but presenting antagonistic effects on the breast. Due to these characteristics, E4 is currently being developed for a number of clinical applications, including contraception and menopausal hormone therapy. Endothelial nitric oxide (NO) is a key player for vascular function and disease during pregnancy and throughout aging in women. Endothelial NO is an established target of estrogens that enhance its formation in human endothelial cells. We here addressed the effects of E4 on the activity and expression of the endothelial nitric oxide synthase (eNOS) in cultured human umbilical vein endothelial cells (HUVEC). E4 stimulated the activation of eNOS and NO secretion in HUVEC. E4 was significantly less effective compared to E2, and a peculiar concentration-dependent effect was found, with higher amounts of E4 being less effective than lower concentrations. When E2 was combined with E4, an interesting pattern was noted. E4 antagonized NO synthesis induced by pregnancy-like E2 concentrations. However, E4 did not impede the modest induction of NO synthesis associated with postmenopausal-like E2 levels. These results support the hypothesis that E4 may be a regulator of NO synthesis in endothelial cells and raise questions on its peculiar signaling in this context. Our results may be useful to interpret the role of E4 during human pregnancy and possibly to help develop this interesting steroid for clinical use.

## Introduction

Estetrol [estra-1,3,5(10)-triene-3,15α, 16α, 17β-tetrol] is an estrogenic hormone discovered in 1965 by Diczfalusy and co-workers at the Karolinska Institute in Stockholm (1, 2). E4 is produced exclusively by the human fetal liver during pregnancy. It is present in fetal blood and reaches maternal circulation through the placenta and can be detected in amniotic fluid and maternal urine (3–6). E4 concentrations increase exponentially during pregnancy and peak at term with fetal levels about 10–20 times higher than in the mother. After delivery, blood levels of E4 become rapidly undetectable (7–9).

The pharmacological properties of E4 have been intensely investigated, but yet the physiological function of E4 has not been clearly understood. For example, in contrast with E2, E4 does not stimulate nor binds to sex hormone-binding globulin (SHBG) (10) and also does not inhibit the activity of cytochrome P450 liver enzymes; it is only minimally metabolized and completely excreted in the urine unaltered (11). E4 has a long half-life in humans, with an average of 28 h, which is about twofold longer than E2 (12).

E4 has a low to moderate affinity for both human estrogen receptor alpha (ER alpha) and ER beta with a four/fivefold preference for ER alpha (11). Based on this relatively low receptor binding affinity compared to E2, E4 was originally thought to be a weak estrogen (13). Subsequently, it was found that in rats, E4 acts as an estrogen on bone, brain, vagina, and endometrium (14, 15). However, it surprisingly acts as an estrogen antagonist on the rat breast DMBA model, where it prevents the development of new breast tumors and stimulates the regression of pre-existing ones (16, 17). The estrogen-antagonistic effect of E4 in the breast has been further supported by the observation of the ability of this steroid to decrease the stimulatory effect of E2 on cytoskeletal rearrangement, horizontal migration, and matrix invasion of ER+ T47-D human breast cancer cells (18).

The role of estrogens on the cardiovascular system has been largely studied. Estrogens promote vasodilatation, endothelial remodeling, and repair and counteract atherosclerosis (19). A key mechanism of action of estrogens in the cardiovascular system stays in the regulation of nitric oxide (NO) synthesis in endothelial cells (20–24).

Nitric oxide is a central controller of vascular function; it is a vasoactive molecule primarily produced by the vascular endothelium that exerts vascular-protective and anti-atherogenic effects on the vessel wall. NO decreases platelet aggregation and adhesion to the endothelium (25), limits vascular smooth muscle proliferation (26), inhibits neointima formation (27), prevents monocyte chemotaxis (28), and inhibits leukocyte adhesion to the endothelium by transcriptionally reducing adhesion molecule expression (29). Endothelial nitric oxide is produced by the endothelial nitric oxide synthase (eNOS) isoform upon conversion of the substrate l-arginine to l-citrulline (30). More recently, NO has been identified as a key agent in maintaining fetal oxygenation through the regulation of feto-placental blood flow. This is true in normal and even more in pregnancies where fetal oxygen delivery fails, such as during fetal growth restriction (31, 32).

We here characterized the effects of E4 on human umbilical vein endothelial cells (HUVEC), and specifically on eNOS activity and NO synthesis. Furthermore, we addressed the possible regulatory role of E4 on E2-related NO synthesis.

## Materials and Methods

### Cell culture and treatments

Human umbilical vein endothelial cells were obtained from umbilical veins from healthy women at the time of delivery. Written informed consent was obtained from each patient in accordance with the declaration of Helsinki. After collection, the umbilical cord was rapidly immersed in sterile saline solution and immediately processed for endothelial cell isolation. Under a laminar flow sterile hood, the umbilical vein was cannulated and thoroughly rinsed with sterile saline solution. After clamping the other extremity, the vein was filled for 30 min with 1 mg/mL type IA collagenase (Sigma-Aldrich, USA) pre-warmed at 37°C. The action of the collagenase solution was blocked with DMEM 10% FBS, the collected solutions were centrifuged at 4°C for 30 min at 1300 rpm, the supernatant was discarded, and the cell pellet was gently resuspended in phenol red-free DMEM (Gibco, Thermo Fisher Scientific) contained 10% FBS (Lonza Walkersville, Inc.), antibiotic–antimycotic (Gibco, Thermo Fisher Scientific), 2 mM l-glutamin (Gibco, Thermo Fisher Scientific), 25 mM HEPES (Gibco, Thermo Fisher Scientific), 0.2 μg/mL human epidermal growth factor (Sigma-Aldrich, USA), and 50 U/mL heparin. The cells were then plated on culture plates pre-coated with 1% sterile gelatin. All cell cultures were maintained in a humidified 5% CO_2_ atmosphere at 37°C and were grown to confluence and used between passage 3 and 4. Endothelial cell purity is extremely high after passage 2, due to elimination of contaminating macrophages and smooth muscle cells through replating. In confluent HUVEC, cell proliferation rate is negligible due to contact inhibition. Cell count and trypan blue exclusion were used to assess in preliminary experiments whether the experimental treatment may result in modified cell proliferation or death rates, and no significant changes were noted. Before experiments, medium was replaced for 24 h with steroid-deprived FBS (Lonza Walkersville, Inc.) and, when the inhibition treatment was required, the active treatments were done 30 min afterwards it. Control cells always received the same amount of ethanol (solvent for E2/E4, 0.01% final concentration). Estetrol was kindly provided by Herjian Coelingh Bennink (Pantarhei Biosciences), 17β-estradiol was from Sigma-Aldrich, USA-Aldrich, and ICI 182,780 from Tocris Cookson, UK.

### Endothelial nitric oxide synthase activity assay

Endothelial nitric oxide synthase activity was determined as conversion of ^3^H-arginine to ^3^H-citrulline in endothelial cell lysates with NOS assay kit (Calbiochem, Merck-Millipore, Germania). Briefly, HUVEC cells were harvested in ice-cold PBS and were pelleted in a microfuge at 13,000 rpm for 10 min at 4°C, then were homogenized in a buffer containing 25 mM Tris–HCl, pH 7.4, 1 mM EDTA and 1 mM EGTA, 1 mM DTT, 10 μg/mL leupeptin, 2 μg/mL aprotinin, and 100 μg/mL PMSF. About 0.001 mCi ^3^H-citrulline was separated with use of an acidic ion-exchange resin, as described in Ref. (33). Extracts incubated with the eNOS inhibitor, 1 mM *N*-nitro-l-arginine methyl ester, were used as blank. Results were expressed as picomoles of converted citrulline per milligram of assayed protein extracts.

### Nitrite assay

Nitric oxide production was determined as the overall amount of stable NO metabolites, nitrites, released in the cell culture medium during treatment. Briefly, the fluorescent product ^1^H-naphthotriazole was measured in the cell culture medium with excitation/emission wavelengths of 365 and 450 nm and was formed by the reaction of nitrosonium cation that forms spontaneously NO and 2,3-diaminonaphthalene (34). Standard curves were constructed with sodium nitrite. Non-specific fluorescence was determined in the presence of 3 mM NG-monomethyl-l-arginine.

### Immunoblottings

After treatments, cells were collected on ice with lysis buffer containing 50 mM Tris–HCl, pH 7.4, 1 mM EDTA, 1% IGEPAL, protease inhibitor cocktail (Sigma-Aldrich, USA), and phosphatase inhibitor cocktail 3 (Sigma-Aldrich, USA). The concentration of total proteins was quantified by Pierce Micro BCA Assay (Thermo Fisher Scientific). Samples, containing 25 μg of protein, were separated on 10% SDS-page gels and transferred to a PVDF membrane (Immobilon-P, Millipore). Antibodies against the following proteins were used: eNOS (Transduction Laboratories, BD Biosciences), Ser1177-p-eNOS (Upstate Biotechnology, USA), Akt and Thr^308^-p-Akt (Upstate Biotechnology), ER alpha (sc-8005, Santa Cruz Biotechnology, USA), and ER beta (sc-390243, Santa Cruz Biotechnology, USA). Blots were blocked in 5% BSA and probed with primary antibody at 4°C overnight, then with an appropriate secondary antibody conjugated to horseradish peroxidase (Santa Cruz Biotechnology, USA) at room temperature for 2 h. Immunodetection of protein bands were visualized using chemiluminescence and were recorded with a quantitative digital imaging system (Quantity One, BioRad, USA). Meanwhile, the membranes were stripped and reproved with anti-actin antibody (Santa Cruz Biotechnology, USA) to confirm equal amounts of loaded samples. Densitometric analysis of the proteins bands was performed using the NIH ImageJ 1.40g software.

### Statistical analysis

Each experimental condition was reproduced in at least three independent experiments. All data are presented as mean ± SEM. Statistical analysis was performed using GraphPad Prism 5. Statistical differences between means were analyzed using one-way ANOVA followed by Bonferroni post-test. Differences at *p* < 0.05 were considered significant.

## Results

### E4 increases eNOS expression/activity and NO synthesis via ERs in HUVEC

**Figure 1:**
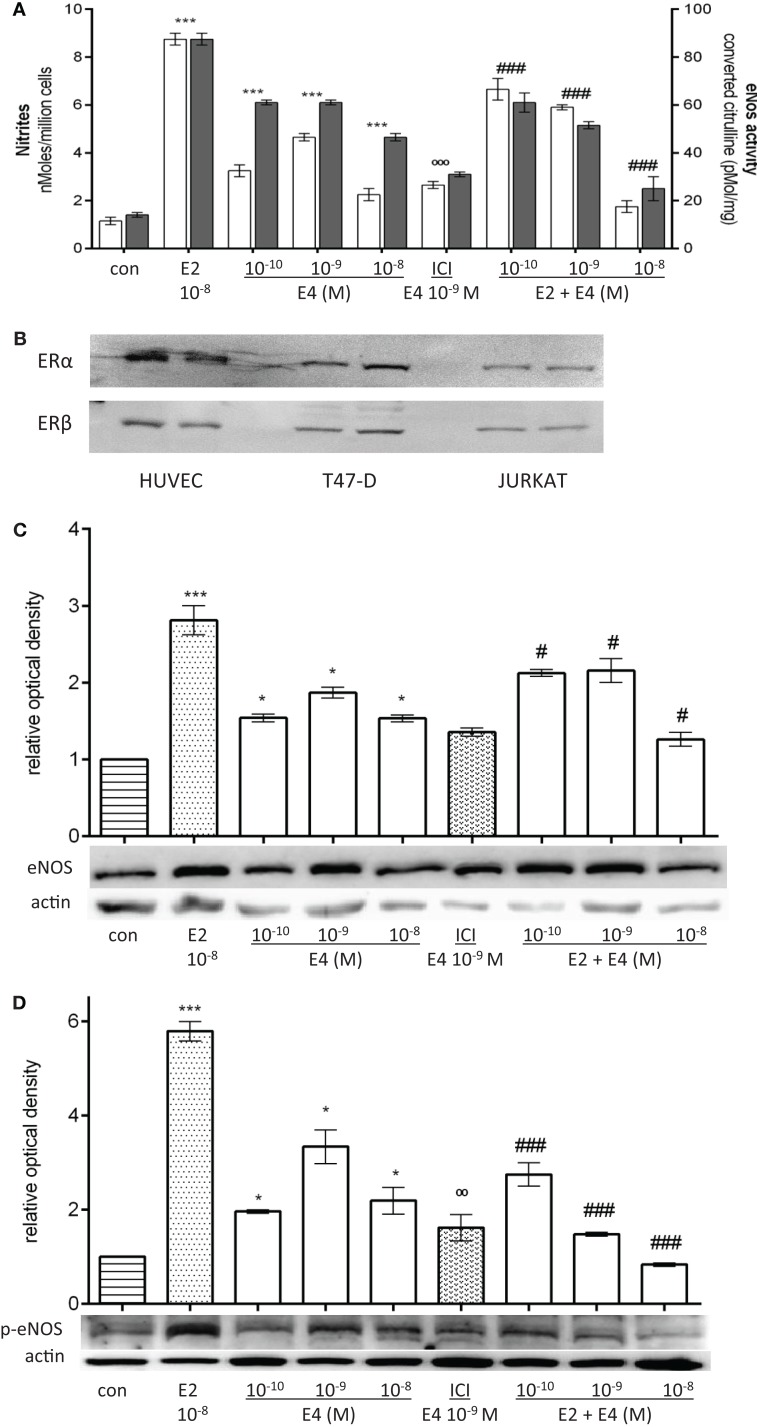
**E4 modulates eNOS during prolonged administration via ER**. Steroid-deprived HUVEC were treated for 48 h with 10^−8^M E2 or increasing concentrations of E4 (10^−10^, 10^−9^, and 10^−8^M) in the presence or absence of the pure estrogen antagonist ICI 182,780 (10^−7^M). **(A)** White bars represent nitrites levels and black bars represent enzymatic activity of eNOS. The experiments were repeated three times with comparable results. **(B–D)** Cell lysates were analyzed by western blotting for ER alpha, ER beta, eNOS, and Ser^1177^-p-eNOS. Quantification expressed as mean ± SEM for three independent experiments (upper panel) and representative blots are shown (lower panel). Values in the graph bar were obtained by the ratio of each protein band versus a loading reference. Significance of the observed effects was evaluated using one-way ANOVA followed by Bonferroni’s *post hoc* test (***p < 0.001, *p < 0.05 versus control, ˚˚˚*p* < 0.001, ˚˚*p* < 0.01 versus E4, ^###^*p* < 0.001, ^#^*p* < 0.05 versus E2).

To test the effects of E4 on endothelial NO level, we measured NO release in the cell culture medium as well as cellular expression and enzymatic activity of eNOS. HUVEC were treated for 48 h, with increasing concentration of E4 alone representing the physiological levels of E4 throughout pregnancy, or in combination with E2. E4 significantly induced the production of NO and eNOS enzymatic activity (Figure 1A). Protein levels of eNOS (Figure 1C) and of its phosphorylated active form (Figure 1D) were also increased during exposure to E4. However, E4 was significantly less effective compared to equimolar amounts of E2 (Figures 1A,C,D). Surprisingly, we did not find a linear relationship between the amount of E4 administered and the response in terms of NO synthesis, eNOS activity or eNOS expression, or phosphorylation. Indeed, the concentration–effect curve of E4 was bell-shaped, with increasing doses resulting in lower stimulation.

When HUVEC were treated with the pure estrogen receptor antagonist ICI 182,780, E4 effects were significantly reduced, suggesting that E4 acts at least in part via estrogen receptor recruitment (Figures 1A,C,D). ER alpha and ER beta are expressed in cultured HUVEC as shown by comparative western analysis with T47-D breast cancer cells and immortalized T lymphocyte Jurkat cells (Figure 1B).

Interestingly, the effects of an early-pregnancy-like concentration of E2 (10^−8^M) on NO synthesis as well as on cellular expression and enzymatic activity of eNOS and p-eNOS were significantly reduced when HUVEC were co-treated with E4 (Figures 1A,C,D). Inhibition of E2-dependent actions was related to the amount of E4 added (Figures 1A,C,D).

### Differential regulatory actions of E4 on E2-induced NO production and eNOS expression based on the relative concentrations of the two steroids

Since in women concentration of E2 varies throughout reproductive life, three different concentrations of E2, mimicking early pregnancy (10^−8^M), follicular phase (10^−9^M), or post-menopause (10^−10^M) were used to study how NO production and eNOS expression are influenced in these three settings by E4. HUVEC were treated for 48 h, with E2 alone or in combination with increasing amounts of E4.

**Figure 2:**
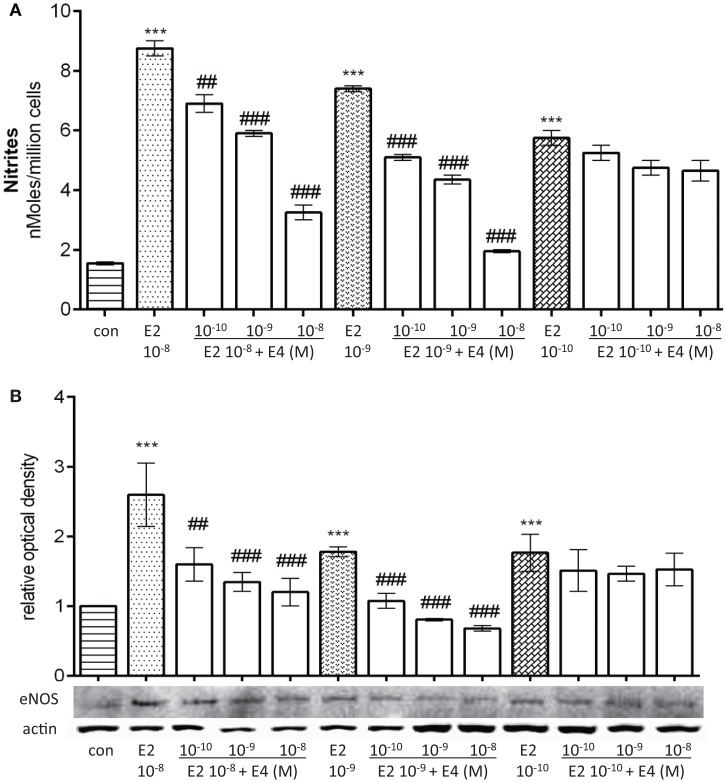
**Differential regulatory actions of E4 on E2-induced NO production and eNOS expression based on the relative concentrations of the two steroids**. Steroid-deprived HUVECs were treated for 48 h with increasing concentrations of E4 and E2. **(A)** Bars represent nitrites levels. The experiments were repeated three times with comparable results. **(B)** Cell lysates were analyzed by western blotting for eNOS. Values in the graph bar were obtained by the ratio of each protein band with a loading reference. Data are expressed as mean ± SEM for three independent experiments. Significance of the observed effects was evaluated using one-way ANOVA followed by Bonferroni’s *post hoc* test (***p < 0.001 versus control, ^###^*p* < 0.001, ^##^*p* < 0.01 versus E2).

Addition of E4 to E2-treated cells resulted in a differential blockade of E2-dependent effects, but the extent of this action varied based on both E2 and E4 amounts. Indeed, when growing amounts of E4 were added to pregnancy-like E2 concentrations, a visible reduction of estradiol-mediated NO synthesis and eNOS expression was found (Figures 2A,B). On the opposite, when E4 was added to postmenopausal-like amounts of E2, a non-significant fall in NO synthesis and a lower reduction of eNOS expression was found (Figures 2A,B). This is particularly interesting, since it suggests that E4 is able to antagonize NO production in the presence of normal to high levels of E2, but not in the presence of low amounts, which deserves an explanation.

### E4 modulates NO synthesis via rapid extranuclear signaling of ERs

Beyond being able to increase eNOS expression through conventional genomic mechanisms, estrogens also enhance NO synthesis through extranuclear-initiated signaling pathways in HUVEC (35–37). To assess whether E4 signaling to ERs also recruits this sort of mechanism, we exposed HUVEC for 30 min to E4 or E2.

**Figure 3:**
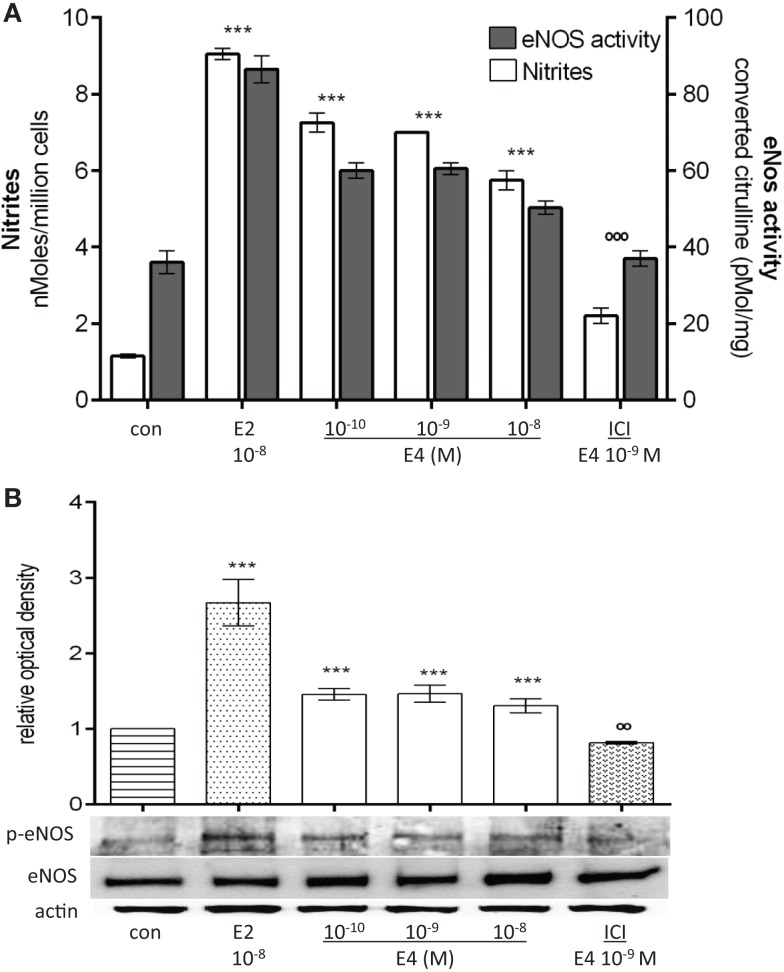
**E4 rapidly modulates NO synthesis and eNOS enzymatic activity**. Steroid-deprived HUVEC were treated for 30 min with 10^−8^M E2 or increasing concentrations of E4 in the presence or absence of the estrogen receptor antagonist ICI (10^−7^M). **(A)** White bars represent nitrites levels in HUVEC and the black bars represent the enzymatic activity of eNOS. The experiments were repeated three times with comparable results. **(B)** Cell lysates were analyzed by western blotting for eNOS and Ser^1177^-p-eNOS. Quantification expressed as mean ± SEM for three independent experiments (upper panel) and representative blots are shown (lower panel). Values in the graph bar were obtained by the ratio of eNOS/p-eNOS protein band intensity. Significance of the observed effects was evaluated using one-way ANOVA followed by Bonferroni’s *post hoc* test (***p < 0.001 versus control, ˚˚˚*p* < 0.001, ˚˚*p* < 0.01 versus E4).

Rapid E4 administration to HUVEC significantly increased NO release in the supernatant along with eNOS enzymatic activity (Figure 3A). During this type of E4 exposure, rapid phosphorylation of eNOS on Ser^1177^ was observed in the absence of any increase of eNOS expression (Figure 3B). When compared to E2, once more, E4 was less potent in this experimental setting (Figures 3A,B). Similar to the previous findings, increasing the concentrations of E4 over a 100-fold range did not turn into further increases in NO synthesis, eNOS activity nor of Ser^1177^-eNOS phosphorylation. On the opposite, a non-significant decrease in NO release and eNOS activity was noted with increasing E4 concentrations.

Addition of ICI 182,780 dramatically reduced the action of E4, suggesting that also the rapid extranuclear actions of E4 occur at least in part via estrogen receptors (Figures 3A,B).

**Figure 4:**
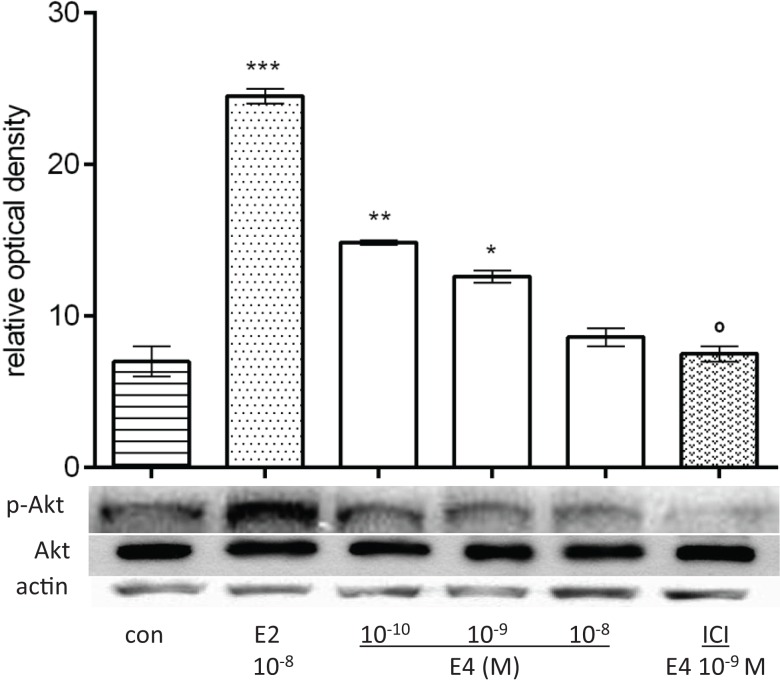
**E4 regulates Akt**. Steroid-deprived HUVECs were treated for 30 min with 10^−8^M E2 or increasing concentrations of E4 (10^−10^, 10^−9^, and 10^−8^M) in the presence or absence of ICI 182,780 (10^−7^M). Cell lysates were analyzed by western blotting for Akt and Thr^308^-p-Akt. Quantification expressed as mean ± SEM for three independent experiments (upper panel) and representative blots are shown (lower panel). Values in the graph bar were obtained by the ratio Akt/p-Akt protein band intensity. Significance of the observed effects was evaluated using one-way ANOVA followed by Bonferroni’s *post hoc* test (***p < 0.001, **p < 0.01, *p < 0.05 versus control, ˚*p* < 0.05 versus E4).

Extranuclear estrogen signaling to eNOS is primarily mediated by the lipid kinase phosphatidylinositol 3-OH kinase (PI3K) and by its downstream effector, the serine–threonine kinase Akt (23, 38–40). We therefore assessed whether E4 also uses this signaling pathway by treating HUVEC for 30 min with E4 or E2. While lower amounts of E4 (10^−10^M) resulted in increased phosphorylation of Akt, further increases in E4 concentrations resulted in a progressive decrease (Figure 4). Akt phosphorylation related to low doses of E4 was decreased by ICI 182,780 suggesting that E4 signaling to Akt relies in part on ER recruitment (Figure 4).

### Differential extranuclear regulatory actions of E4 on E2-induced eNOS and Akt phosphorylation based on the relative concentrations of the two steroids

Similar to the previous set of experiments, we tested whether the modulation by differential combinations of E4 with pregnancy-like, follicular phase-like, or postmenopausal-like E2 amounts were also present when looking at extranuclear signaling to eNOS and Akt phosphorylation.

**Figure 5:**
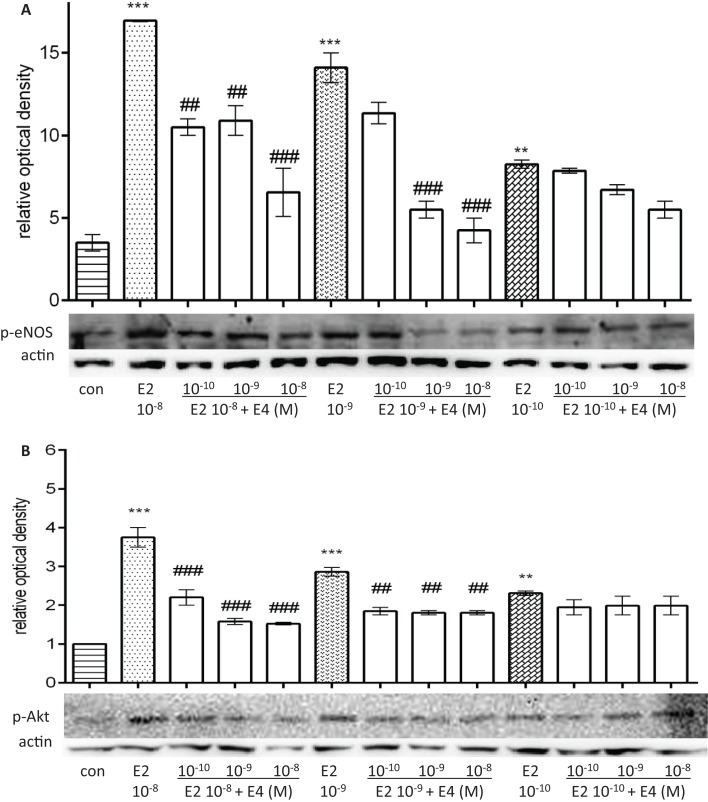
**Differential regulatory actions of E4 on E2-induced eNOS and Akt phosphorylation based on the relative concentrations of the two steroids**. Steroid-deprived HUVECs were treated for 30 min with increasing concentrations of E4 and E2. The total cell lysates were analyzed by western blotting for Ser^1177^-p-eNOS **(A)** and Thr^308^-p-Akt **(B)**. Quantification expressed as mean ± SEM for three independent experiments (upper panel) and representative blots are shown (lower panel). Values in the graph bar were obtained by the ratio of each protein band versus a loading reference. Significance of the observed effects was evaluated using one-way ANOVA followed by Bonferroni’s *post hoc* test (***p < 0.001, **p < 0.01 versus control, ^###^*p* < 0.001, ^##^*p* < 0.01 versus E2).

As shown in Figure 5, E4 decreased in a concentration-dependent manner eNOS and Akt phosphorylation induced by pregnancy-like amounts of E2, while it did not affect phosphorylation of these two targets when co-administered with postmenopausal amounts of E2 (Figures 5A,B).

## Discussion

The main finding of this study is the identification of modulatory actions of estetrol on nitric oxide synthesis in human endothelial cells. E4 controls NO synthesis through seemingly complex signaling avenues. Indeed, the responses to increasing amounts of this steroid are not concentration-dependent. E4 administration to HUVEC turns into increased NO synthesis both because of enhanced eNOS expression and increased enzymatic activation through direct phosphorylation on Ser^1177^. Moreover, estetrol interferes with 17β-estradiol-dependent NO synthesis, but once again it does so with a non-conventional behavior. In the presence of pregnancy-like amounts of E2, E4 blunts NO activation. However, when lower E2 amounts are present, E4 does not interfere with E2 on NO synthesis. These observations may support the hypothesis that E4 could be a naturally occurring selective estrogen receptor modulator, but they also raise questions on its signaling mechanisms.

Estetrol is an interesting estrogen, produced only by the human fetal liver during pregnancy, and its physiological role or its mechanisms of action are still unknown. The present results suggest that regulation of vascular endothelial cells in the feto-placental unit may be a target for this steroid during pregnancy. Activation of endothelial NO synthesis in the placenta, umbilical vessels, and in the fetus is key to promote normal fetal development and to allow fetal adjustment to the stress of delivery (32, 41–43).

E4 concentrations are about 10-fold higher on the fetal side as compared to the maternal one. We here find that E4 results in differential regulation of NO synthesis in endothelial cells based (a) on its concentration and (b) on the amounts of estradiol available. This may suggest that the differential amounts of E4 in the mother and the fetus may result in a selective modulation of the powerful induction of NO synthesis by estradiol at these two sites. The high concentrations of E4 found throughout gestation (ranging between 400 and 1200 pg/mL) may thus represent a pregnancy-specific way to modulate NO production.

If this were true, it may be envisioned that on the maternal side, the lower amounts of E4 result in a lower inhibition of the E2-induced NO synthesis, therefore allowing for efficient vasodilatation toward the fetus. On the contrary, high amounts of E4 on the fetal side may be helpful to prevent excessive vascular dilatation, or possibly stimulation of other estrogen targets, such as the fetal breast. This is consistent with the recent data by Gerard et al. indicating that E4 antagonizes the stimulatory effects of E2 on breast glands in an animal model (44), where the antagonistic actions of E4 are similar in terms of concentration-dependency, to those we find in human endothelial cells.

While E4 is only found in humans during pregnancy, its peculiar chemical structure makes it interesting for exploitation as an endocrine tool in a variety of settings. For instance, its suggested estrogen-antagonistic action in breast tissue, along with its effectiveness in decreasing frequency and intensity of hot flashes (45), in preventing vulvo-vaginal atrophy (15) and osteoporosis (14), makes it attractive as a new form of menopausal hormone therapy. In this setting, the vascular actions of this steroid become particularly relevant. Estrogen replacement therapy has been shown in many studies to exert a vascular-protective action (19, 46), and most of these effects are attributed to the induction of NO synthesis in endothelial cells (35, 36, 47–50). Thus, should E4 result in decreased NO synthesis in endothelial cells, this would be undesirable. To this extent, our results indicate that in the presence of lower estradiol amounts, similar to those found after menopause, E4 does not significantly counteract E2-induced NO synthesis.

Why E4 does not increase eNOS expression and activity or NO synthesis in a linear concentration-dependent manner is unclear. Similarly, it is difficult to interpret why interference with NO synthesis induced by E2 does not happen in the presence of low estradiol concentrations. Given the high degree of similarity of cell responses through which E4 induces NO as compared to E2 (increased eNOS protein expression, increased eNOS activity, increased phosphorylation of eNOS), it would be tempting to attribute the interference of E4 with E2 signaling to competitive inhibition for ERs when the two estrogens are jointly present. To this extent, affinity of E4 for ER alpha is around 100-fold lower than that of E2 (11). Inhibition of ERs with a pure ER antagonist (ICI 182,780) blunts some of the effects of E4, thereby supporting the view that it is at least in part through ER binding that E4 affects NO synthesis in human endothelial cells.

However, this does neither seem to explain the peculiar E4 concentration-related responses of HUVEC in terms of NO nor the non-conventional inhibition of E2-related NO induction. Other signaling mechanisms may thus be involved, such as induction of a shift from the membrane of significant populations of ERs or alternatively an increased internalization and destruction of ERs. In a recent manuscript from Abot et al., E4 has been shown to be able to activate *in vivo* the ER alpha-dependent signaling that mediate protection from atherosclerosis development, while failing to activate the membrane-initiated signaling involved in ER alpha-dependent re-endothelialization and eNOS activation (51). The authors show that the three-dimensional structure of ER alpha ligand-binding domain in the presence of E2 or E4 is very similar, with only a minor shift in the orientation of helix 12 that is not expected to turn into significant modifications in the interaction with ER-interacting proteins (51). Interestingly, in the same manuscript, the authors show that both E2 and E4 are able to trigger the protein–protein interaction of ER alpha with one of its main signaling partners, c-Src. This small adapter protein is the upstream activator of ER alpha-dependent signaling to PI3K and MAPK, and is the key initiator of membrane-initiated estrogen signaling to eNOS (22). In the Abot paper, when E2 and E4 were provided together, ER alpha interaction with c-Src was significantly inhibited below baseline (51). Based on these observations, it is possible that part of the unexpected effects of higher concentrations of E4 or the selective inhibition of ER signaling associated with high but not low E2 concentrations may be ascribed to the ability of E4 to uncouple the recruitment of membrane-initiated signaling through interference with ER alpha/c-Src interaction.

Another potential explanation for the effects of E4 on NO synthesis in HUVEC may also be provided by parallel signaling through other receptors. Recently, Gerard et al. have generated preliminary data supporting the hypothesis that E4 may in part act via the G protein-coupled estrogen receptor 1 (GPER) in breast cancer cells (52). Thus, it is possible that interference with other receptors involved in signaling to eNOS may explain E4 actions in HUVEC. E4 signaling to eNOS in endothelial cells may thus represent a useful model to dissect the signaling mechanisms of this fetal steroid and its biological actions, and will be ground for future investigation.

In conclusion, our present findings that E4 modulates endothelial NO synthesis in HUVEC and that it also interferes with estradiol induction of NO. It is therefore tempting to speculate that E4 may represent a naturally occurring compound designed by nature to control the maternal and fetal vascular actions of estrogens during gestation.

## Author Contributions

MM-G performed the molecular studies, bioinformatics statistical analysis, and drafted the manuscript. MG designed and performed the molecular studies and supervised the project. ER, AG, and PM performed the molecular studies. ARG and ADG supervised the project. TS designed and participated in the data interpretation, helped to draft the manuscript, and supervised the project. All authors read and approved the final manuscript.

## Conflict of Interest Statement

The authors declare that there is no conflict of interest that could be perceived as prejudicing the impartiality of the research reported.
